# Left Atrial Ball-Shaped Thrombus with Concomitant Biatrial Appendage Thrombi in a Patient with Prior Mitral Valve Replacement

**DOI:** 10.1155/2019/6531890

**Published:** 2019-04-16

**Authors:** Kanako Akamatsu, Takahide Ito, Kazushi Sakane, Yumiko Kanzaki, Koichi Sohmiya, Masaaki Hoshiga

**Affiliations:** Department of Cardiology, Osaka Medical College, Takatsuki, Osaka, Japan

## Abstract

We reported a 67-year-old woman in whom large atrial thrombi were found by chance during discontinuation of therapeutic anticoagulation. The patient, with a history of mitral valve replacement surgery, had stopped anticoagulation for months because of intractable gastrointestinal bleeding, during which she was found to have 3 large thrombi in the atria on transesophageal echocardiography: left atrial free-floating ball-shaped thrombus, left atrial appendage thrombus, and right atrial appendage thrombus. One month following diagnosis, she still had the free-floating thrombus despite adequate anticoagulation. Free-floating ball-shaped thrombus is a rare finding observed on echocardiography in patients with mitral valve disease and an even rarer finding in case of appendage thrombi coexisting.

## 1. Introduction

Free-floating ball-shaped thrombus in the left atrium is a rare type of cardiac thrombus that may cause embolism, syncope, congestive heart failure, and sudden death [1–3]. It was first reported by Wood in 1814, who described an autopsy record in a 15-year-old girl with rheumatic mitral stenosis presenting recurrent syncope [3]. Since then, there have been a volume of reports on this entity particularly in the echocardiography era [4–11]. We herein reported a 67-year-old woman with prior mitral valve replacement surgery in whom multiple atrial thrombi, including a free-floating ball-shaped thrombus, were found on echocardiography.

## 2. Case Report

**Figure 1:**
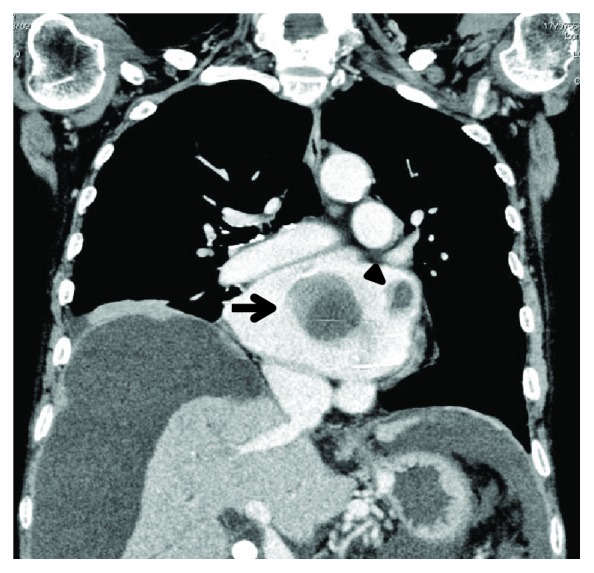
Computed tomography scan of the heart showing the free-floating ball-shaped thrombus (arrow) and appendage thrombus (arrow head). They look blurred due to movement within the left atrium.

The woman was referred to the department of cardiology with consultation for left atrial masses detected by chance with follow-up echocardiography and computed tomography (Figure 1). Eight years ago, the patient had undergone mitral valve replacement surgery for rheumatic mitral stenosis. She had discontinued therapeutic anticoagulation for 4 months because she suffered from intractable gastrointestinal bleeding due to radiation therapy for peritoneal dissemination of uterine cancer. Her electrocardiogram showed atrial fibrillation with a heart rate of 84 bpm. Transthoracic echocardiography revealed a free-floating round-shaped mass in the left atrium that was not detected 5 months before. The left ventricular dimension (44 mm) and ejection fraction (62%) were normal, and the left atrial diameter was markedly increased (65 mm).

**Figure 2:**
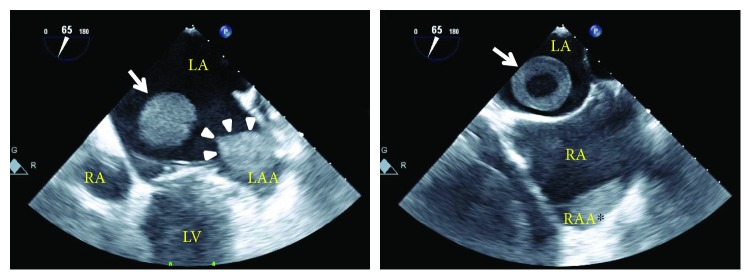
Two-dimensional transesophageal echocardiographic images of the free-floating ball-shaped mass (arrows), left (arrow heads), and right (^∗^) atrial appendage thrombi. LA: left atrium; LAA: left atrial appendage; LV: left ventricle; RA: right atrium; RAA: right atrial appendage.

**Figure 3:**
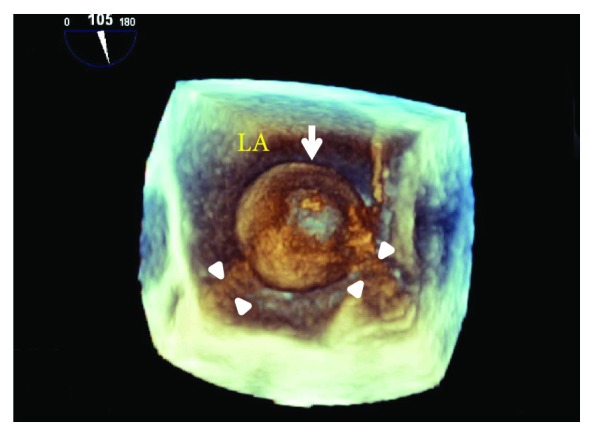
Three-dimensional transesophageal echocardiographic image of the free-floating ball-shaped mass (arrow) around which some sludge (arrow heads) is present suggesting a potential mechanism of its formation. Abbreviations are the same as in Figure 2.

**Figure 4:**
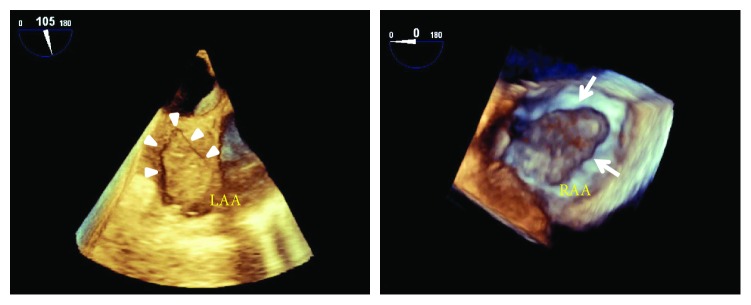
Three-dimensional transesophageal echocardiographic images of the left (arrow heads) and right (arrows) atrial appendage thrombi. Abbreviations are the same as in Figure 2.

With the subsequent evaluation by transesophageal echocardiography, using an x8-2t transducer on an EPIQ 9 ultrasound machine (Philips Medical Systems, Andover, MA, USA), 2 atrial thrombi occupying the left and right atrial appendage cavities, respectively, in addition to the free-floating round-shaped mass were found (Figure 2). The left atrial appendage thrombus was fibrillated with cardiac beats while the right atrial appendage thrombus was immobile. There was a degree of spontaneous echo contrast in both atria indicating a low-flow condition. A moderate degree of stenotic physiology was present across the mitral valve (9.0 mmHg of the mean pressure gradient). With the real-time 3-dimensional transesophageal echocardiographic imaging, the round-shaped mass was ascertained to be ball-shaped (Figure 3). The 3-dimensional images of both appendage thrombi are shown in Figure 4.

Based on her clinical history (long-standing atrial fibrillation and postprosthetic valve implantation), situations (discontinued anticoagulation and coexistence of appendage thrombi), and previous reports on similar cases [4–11], the ball-shaped free-floating mass was implied as a thrombus. Although a surgical procedure for the atrial thrombi was considered, the patient was determined to be managed conservatively with therapeutic anticoagulation, because she was diagnosed as having terminal cancer and thus in a malnourished condition. One month following anticoagulation, she still had the free-floating thrombus on transthoracic echocardiography. She died a few months after moving to another hospital for palliative care.

## 3. Discussion

Free-floating ball-shaped thrombus in the left atrium is a rare finding among patients with mitral valve disease with or without a history of valve replacement surgery [3–8]. This type of thrombus was reported to form in the absence of valve disease and also to recur despite adequate anticoagulation [10, 11]. The precise mechanism behind the occurrence of a free-floating ball-shaped thrombus remains uncertain. In the beginning, the thrombus is considered to be a small mural thrombus attached to the atrial wall [2]. The thrombus gradually grows and projects into the atrial cavity while connecting with the atrial wall via a thin stalk [9]. Then, as the thrombus becomes larger, the stalk becomes thinner and ultimately breaks off the atrial wall. The thrombus, becoming free, moves around in the atrial cavity, polishes itself by the circumjacent structures, and consequently, takes on a smooth, ball-shaped appearance.

Nevertheless, the mechanism for free-floating ball-shaped thrombus formation that we and others proposed above seems to differ from the observation by Wright-Smith et al., who demonstrated that the cut section of the ball-shaped thrombus resected from a patient with mitral stenosis represented a laminated and onionskin appearance with central cavitation [4]. Their findings of the thrombus were similar to ours in 2- and 3-dimensional echocardiographic findings with respect to its smooth surface and cavity forming [4], suggesting that such a thrombus grows to form ball-shaped, rather than attached to the atrial wall.

Because of the intricate clinical situation, the patient could not receive either surgery or thrombolytic therapy, although prompt surgical removal of the thrombus is generally preferred [2]. We restarted anticoagulation concerning about occurrence of complications such as systemic embolization and valve orifice obstruction. Consequently, no serious complications occurred on the patient until hospital discharge, but her clinical condition precluded histological presentation for the free-floating ball-shaped thrombus, which could have delineated an onionskin appearance as reported by Wright-Smith et al. [4].

Although similar cases were abundantly described [4–11], to the best of our knowledge, a free-floating ball-shaped thrombus with coexisting biatrial appendage thrombi imaged on echocardiography was the first reported case. In the only relevant report, Yoshioka et al. introduced a case of left atrial ball-shaped thrombus in conjunction with left atrial appendage thrombus [11]. For our case, in addition, other than anatomical and functional abnormalities of the left atrium, a low-output state associated with stenotic prosthetic valve might facilitate thrombosis in the appendages. Interestingly, compared to the left atrial appendage thrombus, the right appendage thrombus appeared to be organized to a more extent (Figure 4), presumably growing under a laminar blood flow condition specific to the right atrium.

## 4. Conclusion

This is the first report in which ball-shaped free-floating thrombus and biatrial appendage thrombi were coincidentally imaged on echocardiography. In this case, however, surgical removal of the thrombus was given up because of serious clinical conditions of the patient.
